# Teratogenic risk and contraceptive counselling in psychiatric practice: analysis of anticonvulsant therapy

**DOI:** 10.1186/1471-244X-13-234

**Published:** 2013-09-25

**Authors:** Julie Langan, Andrea Perry, Maria Oto

**Affiliations:** 1 Institute of Health and Wellbeing, University of Glasgow, Mental Health and Wellbeing, Gartnavel Royal Hospital, 1055 Great Western Road, Glasgow G12 0XH, UK; 2 ST6 General Adult Psychiatry NHSGG&C, Esteem Service, 60 Mollinsburn Street, Glasgow G21 4SF, UK; 3 ST6 General Adult Psychiatry, Gartnavel Royal Hospital, 1055 Great Western Road, Glasgow G12 0XH, UK

**Keywords:** Anticonvulsant, Valproate, Carbamazepine, Lamotrigine, Topiramate, Women, Child bearing age, Teratogenic potential, Contraception

## Abstract

**Background:**

Anticonvulsants have been used to manage psychiatric conditions for over 50 years. It is recognised that some, particularly valproate, carbamazepine and lamotrigine, are human teratogens, while others including topiramate require further investigation. We aimed to appraise the documentation of this risk by psychiatrists and review discussion around contraceptive issues.

**Methods:**

A retrospective review of prescribing patterns of four anticonvulsants (valproate, carbamazepine, lamotrigine and topiramate) in women of child bearing age was undertaken. Documented evidence of discussion surrounding teratogenicity and contraceptive issues was sought.

**Results:**

Valproate was most commonly prescribed (n=67). Evidence of teratogenic risk counselling at medication initiation was sub-optimal – 40% of individuals prescribed carbamazepine and 22% of valproate. Documentation surrounding contraceptive issues was also low- 17% of individuals prescribed carbamazepine and 13% of valproate.

**Conclusion:**

We found both low rates of teratogenic risk counselling and low rates of contraception advice in our cohort. Given the high rates of unplanned pregnancies combined with the relatively high risk of major congenital malformations, it is essential that a detailed appraisal of the risks and benefits associated with anticonvulsant medication occurs and is documented within patients’ psychiatric notes.

## Background

Anticonvulsants have been used in the management of psychiatric conditions for over fifty years [1,2] particularly in the management of bipolar affective disorder [3]. Semi sodium valproate is used in the treatment of acute mania, as a maintenance therapy and in combination with an antidepressant in bipolar depression [3]. While carbamazepine is recommended by the Scottish Intercollegiate Guidelines Network (SIGN) as an alternative maintenance therapy to lithium, particularly in patients with bipolar II or where lithium therapy is not tolerated or is ineffective [3]. Both valproate and carbamazepine are recognised as human teratogens. The overall risk of major congenital malformation in any pregnancy is 2% and this is increased 2–3 fold in women taking a single anticonvulsant drug (namely valproate or carbamazepine) [4]. Studies, mostly in women with epilepsy indicate that both medications independently increase the risk of major congenital abnormalities when administered in the first trimester. This effect is not thought to be related to maternal seizure activity [5]. Valproate in particular is associated with the highest risk of major and minor congenital abnormality, in particular neural tube defects [6]. There is some evidence that the risk is dose dependent [7]. Valproate is also associated with lower IQ in offspring [8]. Due to these risks, valproate (when used as a mood stabiliser) should not be routinely prescribed to women of childbearing potential (SIGN 2012)(NICE 2006). Carbamazepine is associated with higher rates of neural tube defects, cardiovascular and urinary tract abnormalities [9] and there is some evidence that it reduces birth weight [10], reduces fetal head circumference [11] and reduces gestational age at delivery.

Lamotrigine, a newer anticonvulsant, is recommended by SIGN in acute bipolar depression, as maintenance therapy in individuals stabilised on this medication or where depressive relapse is a greater problem than manic relapse [3]. It was initially developed as an alternative to semi sodium valproate in epilepsy for women of child bearing potential. Although a recent study involving the Australian Register of Antiepileptic Drugs in pregnancy found no significant increased risk of malformations for lamotrigine compared to untreated epileptic pregnancies [12] it is recognised that further studies are required to investigate its teratogenic potential. Topiramate another new anticonvulsant has been used off licence for weight reduction in psychiatric patients. Currently it is not recommended by National Institute for Health and Clinical Excellence (NICE) or SIGN in the management of mood disorders. Its teratogenic potential also warrants further longer term study.

The use of these four medications within psychiatry is further diversifying- with increased use “off licence”. For example carbamazepine and valproate may be used as adjuvants to antipsychotics in schizophrenia- [13-15] and both along with lamotrigine may be prescribed in the management of substance abuse (particularly alcohol withdrawal), personality disorder, as an adjuvant in depression and in patients with cyclothymia or dementia [16]. Although there have been no large scale epidemiology studies specifically looking at prescribing rates of anticonvulsants for psychiatric indications in women of child bearing age, it is estimated that one in twenty women of child bearing age who are in long term contact with mental health services are prescribed mood stabilising drugs (many of which are anticonvulsants) [6,17]. Despite this awareness of the teratogenic effects of these medications amongst psychiatrists may be low.

The British National Formulary (BNF) recommends that women of child bearing potential prescribed valproate, carbamazepine, lamotrigine and topiramate should have contraceptive advice prior to commencing these medications [18] although this is not mandatory. Given that there are no specific local guidelines or inclusion in the SIGN guidelines offering advice surrounding these issues and a negative pregnancy test is not essential prior to commencement; awareness of these issues in psychiatrists may be low leading to low rates of adequate counselling. Indeed a survey of women initiated on either valproate or carbamazepine in the psychiatric department of three teaching hospitals over a twelve month period in England and Wales found that “standards of documentation regarding child bearing issues in this representative sample (was) poor” [19]. Given recent estimates that 50% of pregnancies in the UK are unplanned [20,21] (and it is likely that rates of unplanned pregnancies in the psychiatric population are above population norms [22]) the issues of contraception, teratogenicity counselling and the importance of documenting these discussions, are particularly pertinent to psychiatrists.

## Construct and content

### Aims

We aimed to survey the use of valproate, carbamazepine, lamotrigine and topiramate for psychiatric indications in women of child bearing age in NHS Lanarkshire to determine whether or not counselling by psychiatrists regarding teratogenic risk prior to treatment commencement occurred. We also sought to determine if contraceptive issues had been discussed by the prescribing psychiatrist.

## Method

We undertook a retrospective analysis of all secondary care psychiatric contacts in NHS Lanarkshire, Scotland which comprises a population of 550,000. The electronic records were phased into mental health services over the period 2002–2005. General adult, rehabilitation, liaison, addiction and forensic psychiatry services all use the electronic record system. The electronic record contains all medical outpatient letters, hospital discharge summaries and the majority of clinical contacts between patients and other mental health care professionals. As such it represents an accurate record of all clinical decisions including medication changes that are made. This review was registered as a clinical audit at NHS Lanarkshire’s Research and Development Department (Project ID 3353). Ethical approval was not deemed required.

We searched our electronic records using the keywords “depakote”, “semi sodium valproate”, “valproate”, “epilim chrono”, “carbamazepine”, “tegretol”, “lamotrigine”, “lamictal”, “topiramate” and “topamax”. All females of child bearing age (defined as between 16 and 50) were included. Those with a diagnosis of epilepsy were excluded and all other ICD 10 diagnoses were included. Those with epilepsy were excluded as the medications of interest were not prescribed for a psychiatric indication and not initiated by psychiatrists. Therefore any discussion surrounding contraceptive issues would not be documented in the psychiatric record. Individuals in whom the electronic record was considered inadequate for analysis i.e. those in whom the medication was commenced before the electronic record began were excluded.

All electronic records were then reviewed by JL and AP and the relevant prescribing data was extracted from the clinical correspondence generated at each clinical contact. This method provides a reliable reflection of psychiatrists’ prescribing patterns as medication initiation is captured on the electronic record. In cases, where there was evidence of diagnostic instability our diagnostic stratification was based on the most prominent diagnosis by a consultant psychiatrist. Reason for medication initiation was noted along with maximum drug dosage. Defined daily dose was calculated along with duration of treatment.

Documentation of counselling regarding teratogenic risk at initiation of treatment by the prescribing psychiatrist was sought. Counselling regarding teratogenic risk was defined as any free text comment surrounding teratogenic effects of medication entered into the clinical record at any clinical contact (including both outpatient review and hospital discharge letter). Evidence of discussion surrounding contraceptive issues was also sought by searching for a free text comment surrounding this issue again at any clinical contact. Any pregnancies at medication initiation or subsequent to this were documented.

## Results

The average number of referrals (per year) over the last 5 years for females aged between 16 and 50 to general adult psychiatry in NHS Lanarkshire was 1,260.

**Figure 1:**
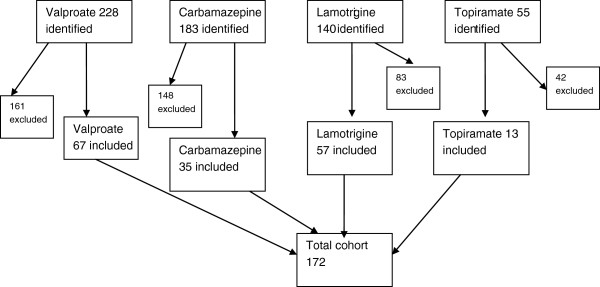
Identification of the cohort of women prescribed medications of interest.

606 electronic records containing our key words were identified. 434 records were excluded due to a combination of inadequacy of record, non psychiatric indication for medication use (e.g. epilepsy) and women not actively prescribed the medication (See Figure 1). A cohort of 172 patients was then identified. Valproate was the most commonly prescribed anticonvulsant (67/172, 40.0%), followed by lamotrigine (57/172, 33.1%), carbamazepine (35/172, 20.3%) and topiramate (13/172, 7.6%).

The average age of females prescribed the medications of interest was similar at 39.7 years as was the age range (20 to 50) [Table 1]. Average duration of treatment was greater than 1 year, although the range in duration of prescription was variable, with carbamazepine being prescribed for 49 months on average [Table 1].

**Table 1:** Clinical details of the anticonvulsants prescribed

	**Valproate (n=67)**	**Carbamazepine (n=35)**	**Lamotrigine (n=57)**	**Topiramate (n=13)**
Average Age years (range)	39.3 (23.6-50.0)	39.6 (20.2-50.0)	39.1 (20.2.-50.0)	40.7 (28.1-48.5)
Average duration of treatment months (range)	27 (1–91)	49 (1–238)	32 (1–90)	14 (1–62)
ICD 10 Diagnosis				
Bipolar Affective Disorder % (n)	39 (26)	37 (13)	37 (21)	46 (6)
Unipolar Depression % (n)	21 (14)	43 (15)	49 (29)	38 (5)
Schizoaffective Disorder % (n)	10 (7)	0 (0)	5 (3)	0 (0)
Schizophrenia % (n)	8 (5)	0 (0)	2 (2)	0 (0)
Emotionally Unstable PD % (n)	10 (6)	6 (2)	0 (0)	8 (1)
Cyclothymia % (n)	8 (5)	3 (1)	2 (1)	0 (0)
Other % (n)	6 (4)	11 (4)	2 (1)	8 (1)
Mean dose of medication/day mg/day (range)	987 (100–2000)	455 (200–800)	166 (25–300)	192 (45–400)
Mean Dose/day (DDD)^1^	0.66	0.46	0.55	0.65

^1^*DDD* Defined Daily Dose, 1DDD is equivalent to valproate =1500 mg, Carbamazepine = 1000 mg, Lamotrigine = 300 mg & Topiramate = 300 mg [23].

For all anticonvulsants a mood disorder was the most common ICD 10 diagnosis- with valproate and topiramate most commonly being prescribed in patients with bipolar affective disorder. Prescription of the anticonvulsants as an augmentation to anti-depressant medication in unipolar depression was a commonly used strategy in our cohort and was the most common indication for the use of carbamazepine and lamotrigine Unlicensed use occurred relatively frequently in our cohort, including as an adjunctive in the management of schizophrenia, schizoaffective disorder, emotionally unstable personality disorder and cyclothymia [Table 1].

Evidence of counselling by the prescribing psychiatrist regarding teratogenic risk was low amongst all four anticonvulsants studied. Counselling rates were highest for carbamazepine (40% n=14) followed by valproate (22% n=15). When teratogenic risk was discussed, contraceptive issues were then discussed in 67% (10/15) of women on valproate and 43% (6/14) of women on carbamazepine. There was no documented evidence of counselling for those prescribed lamotrigine or topiramate [Table 2].

**Table 2:** Rates of counselling surrounding teratogenicity & contraception

	**Valproate (n=67)**	**Carbamazepine (n=35)**	**Lamotrigine (n=57)**	**Topiramate (n=13)**
Evidence of pre-treatment teratogenic counselling % (n)	22 (15)	40 (14)	0 (0)	0 (0)
Documentation of discussion of contraceptive issues % (n)	13 (10)	17 (6)	0 (0)	0 (0)
Number of pregnancies	1	0	4	0

Rates of documentation of discussion regarding contraceptive issues by the prescribing psychiatrist were low occurring in 17% (n=6) of cases prescribed carbamazepine and 13% (n=10) of cases prescribed valproate. There was no documented evidence of discussion regarding contraceptive issues for those prescribed lamotrigine or topiramate [Table 2]. All women, in whom contraception was discussed, had had a discussion with their psychiatrist around teratogenic potential of their medication.

There was no evidence of a negative pregnancy test being required to initiate treatment in any cases in any of the four anticonvulsants. There were five pregnancies documented in our cohort- one occurring while on valproate with subsequent termination (reason unclear) and four in women prescribed lamotrigine- with one termination (reason unclear) and three term live singleton births. In these five cases, risk of teratogenicity was discussed in only one case (when valproate was prescribed). Contraception had not been discussed in any of the cases.

## Discussion and conclusions

Carbamazepine, valproate, lamotrigine and topiramate were prescribed for a variety of psychiatric indications in our cohort of patients. Valproate although most commonly used in the treatment of bipolar affective disorder was also initiated in the management of other conditions most frequently unipolar depression, schizoaffective disorder and emotionally unstable personality disorder. Carbamazepine and lamotrigine too were used in the management of unipolar depression while topiramate was most frequently prescribed in the management of bipolar affective disorder. Our findings are in keeping with clinical suspicion that these medications are used for a diverse range of clinical indications and on an offline licence basis not infrequently.

The average age of women prescribed all four medications in our cohort was similar at 39.7 years. This is older than the 2011 Scottish average age of all women giving birth which was 29.7 years [22]. The age range of women prescribed these medications in our cohort was wide and the average duration of treatment of medications was at least 14 months, with carbamazepine typically being prescribed for the longest duration at 49 months. This long duration of exposure combined with a recent trend towards older age at childbirth in Scotland is of clinical relevance due to the potential risk of an unplanned pregnancy occurring during such a long period of exposure.

Documentation of counselling regarding teratogenic effects and discussion of contraceptive issues by the prescribing psychiatrist occurred at a low rate (<50%) in our population studied. All women who had evidence of discussion surrounding contraceptive issues by their psychiatrist had evidence of counselling of the teratogenic potential of the medication. However not all women who were made aware of the teratogenic potential of their medication had contraceptive issues directly discussed by their psychiatrist. At medication initiation these important issues were not always discussed. This finding is similar to that found by Wieck et al^20^ who looked specifically at valproate and carbamazepine. In our cohort there was a higher rate of counselling for women prescribed carbamazepine compared to all other anticonvulsants including valproate. The reasons for this are unclear, but are of note given valproate’s higher teratogenic potential. The enzyme inducing effect and so risk of contraceptive failure with carbamazepine had been acknowledged in half of cases where contraceptive issues were discussed. We found no clear age trend in counselling rates in our cohort and this may be due to overall small numbers who received counselling. We are unaware of any published reports concerning the documentation of contraceptive and teratogenicity advice by psychiatrists provided to women of child bearing potential prescribed lamotrigine or topiramate and so our study is novel and of clinical interest. Given our findings and in order to raise awareness of these issues within our health board, results of our survey were presented to clinicians at the local regional teaching and an information leaflet for Mental Health Professionals has been developed in association with pharmacy.

Our data appears to indicate that the prescribing psychiatrists do not give sufficient advice surrounding these issues. The reasons for this are likely multi-factorial. It is often difficult to discuss issues surrounding longer term complex medication side effects when patients are acutely unwell and this may partly explain the relatively low rates seen. However not all medications were started in an inpatient setting, which may be a reflection of severity of illness, and a significant proportion of medication prescribed, were initiated in the community at an outpatient appointment. Another possible explanation for the low rates of documentation seen could be that although clinicians raise these topics during their consultation, no written record is made of this. This may raise issues medico-legally, for example if a pregnancy with a congenital malformation was to occur and there is a lack of documentation by the prescribing psychiatrist surrounding teratogenic risk and contraception in the clinical record.

Our methodology was limited by a number of factors. Data extraction using only electronic records, may lead to data being missed as information may be recorded elsewhere such as CPN notes or other clinical paper notes. The electronic record is used by clinicians for all outpatient clinical contacts and discharge letters and should represent an accurate record of all clinical decisions made, including medication changes, potential side effects and any other relevant drug related effects- however data may have been omitted from this record. We also looked only at discussion of teratogenicity and contraception at medication initiation and not at subsequent clinical contacts and so may represent a further limitation. The small numbers of medications studied and a relatively small cohort of prescribing psychiatrists may mean that our results are influenced by independent prescribing patterns and habits of psychiatrists. However given the similarity of our results to others published in this field, we feel that our study is reflective of wider practice. Teratogenic risk may have been discussed by other health professionals including GPs and therefore recorded out-with the electronic record. GPs as the signatory of the prescription may independently discuss these issues with their patients before dispensing and record it in their primary care database. However the purpose of our study was to determine if the prescribing psychiatrist discussed and documented this risk this.

Regardless of the reason for low rates of documentation it is clear that given the prevalence of anticonvulsant medication prescription, combined with high rates of unplanned pregnancies and the relatively high risk of major congenital malformations associated with their use, it is imperative that a detailed discussion regarding the appraisal of the risks and benefits associated with initiation of these medications occurs and is documented within the patient’s psychiatric notes.

## Competing interests

The authors have no competing interests.

## Authors’ contributions

JL- 1st author, lead data collector & corresponding author. AP- 2nd author & 2nd data collector. MO - 3rd author, 3rd data collector & supervisory registrar. All authors read and approved the final manuscript.

## Authors' information

Julie Langan is a Clinical Lecturer working at the University of Glasgow and in NHS Greater Glasgow & Clyde.

Andrea Perry is a ST6 General Adult Psychiatrist currently working in ESTEEM in North Glasgow.

Maria Oto is a General Adult Psychiatrist working within NHS Greater Glasgow & Clyde. She is a trained epileptologist and has multiple research interests including epilepsy, sleep disorder and psychopharmacology.

## Pre-publication history

The pre-publication history for this paper can be accessed here:

http://www.biomedcentral.com/1471-244X/13/234/prepub
